# The clinical features of papillary thyroid cancer in Hashimoto’s thyroiditis patients from an area with a high prevalence of Hashimoto’s disease

**DOI:** 10.1186/1471-2407-12-610

**Published:** 2012-12-21

**Authors:** Ling Zhang, Hui Li, Qing-hai Ji, Yong-xue Zhu, Zhuo-ying Wang, Yu Wang, Cai-ping Huang, Qiang Shen, Duan-shu Li, Yi Wu

**Affiliations:** 1 Department of Head & Neck Surgery, Fudan University Cancer Hospital/Center, Department of oncology, Fudan University Shanghai Medical College, 270 Dong An Road, Shanghai, 200032, PR China

**Keywords:** Papillary thyroid carcinoma, Thyroid-stimulating hormone, Hashimoto’s thyroiditis

## Abstract

**Background:**

The goal of this study was to identify the clinicopathological factors of co-existing papillary thyroid cancer (PTC) in patients with Hashimoto’s thyroiditis (HT) and provide information to aid in the diagnosis of such patients.

**Methods:**

This study included 6109 patients treated in a university-based tertiary care cancer hospital over a 3-year period. All of the patients were categorised based on their final diagnosis. Several clinicopathological factors, such as age, gender, nodular size, invasive status, central compartment lymph node metastasis (CLNM) and serum thyroid-stimulating hormone (TSH) level, were compared between the various groups of patients.

**Results:**

There were 653 patients with a final diagnosis of HT. More PTC was found in those with HT (58.3%; 381 of 653) than those without HT (2416 of 5456; 44.3%; p < 0.05). The HT patients with co-occurring PTC were more likely to be younger, be female, have smaller nodules and have higher TSH levels than those without PTC. A multivariate analysis indicated that the presence of HT and higher TSH levels were risk factors for a diagnosis of PTC. In the PTC patients, the presence of HT or another benign nodule was a protective factor for CLNM, whereas no significant association was found for TSH levels.

**Conclusion:**

PTC and HT have a close relationship in this region of highly prevalent HT disease. Based on the results of our study, we hypothesise that long-term HT leads to elevated serum TSH, which is the real risk factor for thyroid cancer.

## Background

Thyroid cancer is the most common endocrine carcinoma, and papillary thyroid carcinoma (PTC) is the 7^th^ most common cancer among women in the world [1]. There are numerous risk factors for PTC, including radiation exposure, insufficient iodine intake, hormonal factors, and family history [2]. It is noteworthy that molecular analyses have indicated that lymphocytic infiltration is frequently observed in PTC, suggesting that immunological factors might be involved in tumour progression [3,4]. Hashimoto’s thyroiditis (HT) is the most common inflammatory thyroid disease and the most common cause of hypothyroidism in the United States, affecting 22 of every 100,000 individuals. In China, the rates are even higher, with approximately 0.4-1.5% of the population affected, which accounts for 20-25% of the thyroid disease cases in this country [5-7].

Since Dailey et al. first proposed a relationship between HT and PTC in 1955 [8], there have been many reports on this topic, with conflicting conclusions. Certain authors have agreed that there may be a positive correlation between HT and PTC [8-14], whereas others have not [15-19].

We performed a retrospective analysis of the relationship between HT and PTC using a large population sample from an area with a high HT prevalence.

## Results

### Clinical characteristics

In total, 6109 patients underwent thyroid surgery in our hospital during the study period (538 males and 4571 females); 3288 of these patients had a final diagnosis of benign thyroid nodule, and 2821 had a final diagnosis of PTC. The ages of the patients ranged from 8 to 88 years of age (mean, 46.1±12.3 years of age). There were 653 patients with HT in our study population. Age, female gender, and nodule size were significantly different between the patients with and without HT. The basic characteristics of the patients are shown in Table 1. Of the 6109 patients, 5115 patients had had their preoperative serum thyroid-stimulating hormone (TSH) and free thyroxine (T_4_) concentrations measured within the 2 weeks before their surgery and were included in the TSH analysis. Due to the unavailability of their preoperative thyroid function, 994 patients were excluded from the TSH analysis. Among these patients, 260 were prescribed levothyroxine (LT_4_) preoperatively, and the remainder did not have a serum thyroid function test within the 2 weeks prior to their surgery. The 994 patients included 25 patients with HT (23 cases with co-occurring PTC and 2 with benign disease) and 969 patients without HT (178 patients with PTC and 791 with benign disease). All the 5115 patients fulfilled the following criteria: 1) They were not taking L-T4 or methimazole and were not overtly hyperthyroid or hypothyroid. 2) They had TSH, free thyroid hormones and thyroidglobulin antibodies measured within one week prior to thyroidectomy. 3) They had not undergone any type of previous thyroid surgery.

**Table 1:** General status

	**With Hashimoto’s**	**Without Hashimoto’s**	**P value**
**Number of patients**	653	5456	
**Age (years)**	44.4±11.9	46.3±12.3	0.077
**Gender**			
**Male**	56	1482	0.001
**Female**	597	3974	
**Nodule Size (cm)**	1.75±1.23	2.26±1.48	0.001
**PTC**	381(58.3%)	2440(44.3%)	0.001
**PTC cancer with serum TSH**	358(54.8%)	2262(41.6%)	0.001

We then analysed the serum TSH levels of the 5115 patients in the TSH analysis to determine the association between PTC and HT.

### Comparison of the clinicopathological factors between patients with and without HT

In the first phase of the study, we compared the clinicopathological factors of the patients with and without HT. A total of 628 patients with HT and 4487 patients without HT had a serum TSH examination before their surgery. In comparing the patients with and without HT, it was found that gender (female), age, nodule size and serum TSH levels were significant indicators of a positive HT diagnosis. Subsequently, the following factors were compared between the 2 sub-groups: 1) if the patients with HT suffered from PTC; and 2) if the patients with benign disease suffered from HT. The results show that in the patients with HT, both with and without PTC, the patient’s age, nodule size and serum TSH level still were significant indicators of a positive HT diagnosis (made a difference), whereas the gender (female) was not. In the patients with benign disease, both with and without HT, the patient’s gender (female), age, nodule size and serum TSH level also were significant indicators of a positive diagnosis (Table 2).

**Table 2:** Comparison of the clinicopathological factors in patients with or without HT

	**Total patients (5115)**			**Patients with HT (628)**			**Patients with Benign disease(2495)**		
	**With HT**	**Without HT**	**P value**	**HT with PTC**	**HT without PTC**	**P value**	**Benign with HT**	**Benign without HT**	**P value**
**Number of patients**	628(12.28%)	4487(87.72%)		358(57.00%)	270(43.00%)		270	2225	
**Age (years)**	44.35±11.99	46.28±12.27	0.001	42.64±12.04	46.62±11.56	0.001	46.62±11.56	48.38±12.35	0.018
**Female**	573	3248	0.001	330	243	0.339	243	1608	0.001
**Nodule size (cm)**	1.81±1.23	2.32±1.50	0.001	1.21±0.90	2.44±1.32	0.001	2.64±1.32	3.33±1.34	0.001
**TSH**	2.82±2.38	2.22±1.90	0.001	3.26±2.54	2.23±2.02	0.001	2.23±2.02	1.91±2.03	0.025

*:All the patients in the table had serum TSH test.

### Comparison of the clinicopathological factors among patients with PTC

In the second phase of the study, we examined the clinicopathological factors, including HT, in the patients with PTC. Among the patients with PTC, both with and without HT, it was found that gender (female), nodule size, central compartment lymph node metastasis (CLNM) and serum TSH level had a significant difference. Furthermore, we compared the patients with both PTC and HT with the patients with PTC only and the patients with PTC and co-occurring benign diseases. The results show that age, gender (female), CLNM and serum TSH level all differed in the two comparisons. The nodule size only differed in the comparison between the patients suffering from PTC with HT and patients suffering from PTC with benign diseases. The invasion of a primary tumour only differed in the comparison between the patients with PTC and co-occurring HT and patients with PTC only (Table 3).

**Table 3:** Comparison of the clinicopathological factors in PTC patients with TSH

	**PTC with HT**	**PTC without HT**	**P value**	**PTC with HT**	**PTC only**	**P value**	**PTC with HT**	**PTC with Benign**	**P value**
**Number of patients**	358(13.67%)	2262(86.34%)		358(13.67%)	1270(48.47%)		358(13.67%)	992(37.86%)	
**Age (years)**	42.64±12.04	44.21±11.83	0.058	42.64±12.04	44.29±11.73	0.020	42.64±12.04	44.12±11.95	0.023
**Female**	350	1640	0.001	350	925	0.000	350	715	0.001
**Nodule size (cm)**	1.21±0.90	1.33±0.81	0.001	1.21±0.90	1.23±0.78	0.845	1.21±0.90	1.45±0.84	0.001
**Invasion**	69	506	0.250	69	342	0.003	69	164	0.178
**Level VI**	153	1358	0.001	153	827	0.001	153	531	0.001
**Lateral**^&^	37	349	0.133	37	216	0.635	37	133	0.427
**TSH**	3.26±2.54	2.53±1.72	0.001	3.26±2.54	2.45±1.59	0.001	3.26±2.54	2.63±1.86	0.001

* total number of PTC patients with TSH : 2620.

^&^:Lateral neck metastasis ^&.^

### Independent risk factors for PTC

After the two phases discussed above, the clinicopathological factors were examined in univariate and multivariate analyses to assess the PTC risk factors in all 5115 patients. In the univariate analysis, it was found that age (>45 years of age) and nodule size (>1 cm) had significant odds ratios (ORs) less than 1, whereas HT and serum TSH had significant ORs greater than 1. In the multivariate analysis, these four factors were also significant. Interestingly, the HT had an OR of less than 1 after the multivariate analysis (Table 4).

**Table 4:** Independent risk factors for papillary thyroid cancer, as identified by the univariate and multivariate logistic regression analyses

	**Unadjusted OR (95% CI)**	**P value**	**Adjusted OR (95% CI)**	**P value**
**Gender (Male vs. Female)**	1.054 (0.930-1.196)	0.410	0.927 (0.796-1.080)	0.329
**Age (≤45years vs. >45 years)**	0.520 (0.465-0.581)	0.001	0.505 (0.442-0.576)	0.001
**HT (HT vs. no HT)**	1.304 (1.102-1.544)	0.002	0.859 (0.724-0.998)	0.001
**Nodule size (>1 cm vs. ≤1 cm)**	0.010 (0.006-0.016)	0.001	0.009 (0.005-0.014)	0.001
**TSH (constant)**	1.303 (1.254-1.355)	0.001	1.361 (1.304-1.421)	0.001

### Independent risk factors for level VI lymph node metastasis

Because prophylactic central lymph node dissection (CLND) was routinely performed for PTC patients at our hospital, univariate and multivariate analyses of the CLNM were also performed. The results of the univariate analysis showed that PTC patients with HT and PTC patients with benign disease had a significant OR of less than 1, whereas nodule size (>1 cm), serum TSH level and invasion by the primary tumour had significant ORs greater than 1. In the multivariate analysis, the PTC patients with HT and PTC patients with benign disease also showed a protective effect, whereas only nodule size (>1 cm) and invasion by the primary tumour were associated with increased risk (Table 5).

**Table 5:** Independent risk factors for central lymph node metastatic papillary thyroid cancer, as identified by univariate and multivariate logistic regression analyses

	**Unadjusted OR (95% CI)**	**P value**	**Adjusted OR (95% CI)**	**P value**
**Gender (Male vs. Female)**	0.865 (0.722-1.037)	0.117	0.932 (0.763-1.139)	0.493
**Age (≤45years vs. >45 years)**	1.079 (0.923-1.261)	0.341	1.110 (0.933-1.319)	0.239
**HT (HT vs. no HT)**	0.497 (0.396-0.623)	0.001	0.450 (0.347-0.583)	0.001
**Nodule Size (>1 cm vs. ≤1 cm)**	2.507 (2.138-2.941)	0.001	2.332 (1.944-2.797)	0.001
**TSH (constant)**	1.303 (1.254-1.355)	0.001	1.008 (0.962-1.057)	0.727
**With benign (with vs. without)**	0.814 (0.696-0.952)	0.001	0.670 (0.559-0.802)	0.001
**Invasion (yes vs. no)**	9.804 (7.346-13.084)	0.001	10.092 (7.382-13.797)	0.001

### Histomorphological features of HT and PTC

Based on all of the pathological results of the patients in our study population, four typical figures were chosen to show the histomorphological features of HT only, PTC only and HT co-existence with PTC. Additional file 1: Figure S1 shows the histomorphological features of HT, which consisted of the typical germinal centres of HT. Additional file 1: Figure S2 and Figure S3 show the histomorphological features of HT co-existence with PTC. These two figures show the close histomorphological relationship between HT and PTC. Additional file 1: Figure S4 shows the typical histomorphological features of PTC.

## Discussion

Both HT and PTC have a high prevalence worldwide, and these conditions may have a close relationship. Thus, there may be many patients who have both diseases simultaneously. Whether PTC represents a reactive response to HT or HT is a prerequisite tumourigenic event still remains to be determined.

In our population, 653 patients suffered from HT during the period from 2008 to 2010. Of these patients, 58.3% had co-occurring PTC, which was a higher rate than in previous reports [8-19]. If the patients were subdivided by gender, the result was statistically significant for the females with HT. Compared with the females without HT, the women with HT were found to be approximately 30% more likely to have co-occurring PTC. Our findings in this patient population support the previous studies linking HT with PTC [10,13,20].

At the molecular level, the evidence shows that HT may be a precursor of PTC. It is well known that RET was the first activated receptor-tyrosine kinase to be identified in papillary thyroid cancer. The proto-oncogene, located on chromosome 10q11.2, encodes a transmembrane receptor-tyrosine kinase with four cadherin-related motifs in the extracellular domain [21]. The reported overall prevalence of RET/PTC rearrangements in papillary carcinomas varies from 3% to as high as 85%, depending on the detection methods and geographical location of the patients; a reasonable range is 13–43% [22-27]. Moreover, RET/PTC rearrangements have also been reported in the thyroids of patients with the autoimmune inflammatory condition known as Hashimoto’s thyroiditis [28-31]. However, these findings are controversial and might reflect either PCR contamination or the presence of microscopic nodules of papillary carcinoma, as studies that have excluded these possibilities do not confirm the data [32,33]. Whether RET/PTC rearrangements induce HT to PTC remains unknown. The connection of RET/PTC rearrangements in HT and PTC requires further investigation.

However, several retrospective and prospective studies have found no correlation between these two diseases. Interestingly, our univariate analysis found that HT is a risk factor for PTC, whereas HT played a protective role in the multivariate analysis, which is consistent with previous reports. It is difficult to explain this phenomenon. It has been hypothesised that serum TSH levels may play an important role. Since Boalart et al. found that elevated serum TSH can be a risk factor for PTC [34], many studies have replicated their results [15,35-41]. Thus, when considering whether HT is a risk factor for PTC, it is important to evaluate the serum TSH level. In our study, most of the patients’ serum TSH levels were evaluated before their surgeries. As Table 2 and Table 3 show, the HT patients had higher TSH levels than the patients without HT. Moreover, the patients with co-occurring HT and PTC had the highest serum TSH levels among all of the sub-groups.

It is well known that serum TSH has a trophic effect on follicular-cell-derived thyroid cancer growth. Unfortunately, the present study could not provide evidence concerning the malignant transformation of thyroid follicular cells in the TSH-cAMP pathway. Recently, genome-wide association studies (GWAS) and carefully designed candidate gene approaches have determined that the FOXE1 genetic variant, which is downstream of the TSH-cAMP pathway, is the suspected risk factor for follicular-cell-derived thyroid cancer [42-44]. In the future, genetic studies may provide stronger evidence supporting the theory that high TSH levels cause thyroid cancer. When considering HT, it should be recognised that long-term HT can cause hypothyroidism, which elevates the serum TSH level. In China, where iodine intake is increasing, there is a high prevalence of HT and hypothyroidism in subjects with euthyroidism [7]. In our study, the rates of co-occurring PTC in the HT patients were higher than those in previous reports [8-19]. Considering these results, we hypothesise that long-term HT causes elevated serum TSH, which is the real thyroid cancer risk factor. This hypothesis may explain why HT was a risk factor in the univariate analysis, whereas in the multivariate analysis, which controlled for TSH levels, HT became a protective factor. Furthermore, this hypothesis may explain why several of the prospective studies that have examined the association between HT and thyroid cancer have found negative results (i.e., because the HT patients in these studies did not have high serum TSH levels for a long period). Since TSH is a growth factor for PTC, the serum TSH could be an individual marker for each HT patient. In clinic, doctors could control the serum TSH level under a lower level in the normal range of TSH for HT patients [45].

With regard to the clinical course, thyroid tumours associated with HT do not appear to be any more aggressive than those not associated with HT [20,46]. In our series of studies, we found that PTC patients with HT had less aggressive tumours than the patients with PTC only. Interestingly, the PTC patients with HT had less CLNM than those without HT (Table 3). This result conflicts with those of a previous report [14]. In Table 5, TSH is not a factor associated with an increased risk of central lymph node metastases in patients with PTC, which is not in agreement with Kim’s study [47]. The Kim’s study included only 554 patients, which was less than our patients. This might cause our different result. Furthermore, we take TSH as a constants variable, while Kim’s study considered TSH as a dichotomous variable.

Recently, Hanahan et al. proposed a new hallmark of cancer genesis: “Tumor-Promoting Inflammation.” [48] This new hallmark could play an important role in the pathogenic process of co-occurring PTC and HT. In certain cases, it is evident that inflammation is present at the earliest stages of neoplastic progression and capable of fostering the development of incipient neoplasia into cancer and causing the invasion and metastasis of existing cancer [49,50]. It is certain that in the near future, the relationship between inflammation and cancer invasion or metastasis will be further elucidated.

Consistent with previous reports [14,15], our study also found that a nodule >1 cm plays a protective role in our study population (Table 4). This finding may have been due to the selection of patients for surgery. Patients with the small benign nodules are typically not sent to surgery, whereas patients with larger benign nodules, especially >4 cm, are more likely to be candidates for surgical treatment. However, in PTC patients, a primary tumour > 1 cm is a risk factor for neck metastasis (Table 5). Also, there was an interesting phenomenon that the cancer size of “PTC with benign disease” is larger than “PTC only” or “ PTC with HT”, while “PTC with benign diseas” shows less or similar invasion. This might be due to the different genetic origin of different types of PTC. The genesis of "PTC with benign nodule" might come from the benign nodule, which is less invasion [51]. The genesis of “PTC only” might come from the somatic mutation, which is more invasion.

In the morphological examination, an extensive relationship was shown between HT and PTC (Additional file 1: Figure S1-S4). Thus, based on the histomorphological features, laboratory findings and clinical data, the co-occurrence of PTC and HT is an interesting topic concerning thyroid cancer research and cancer hallmarks.

We also have some limitations in this study. Since our study is a retrospective cross-section study, there could be a selection bias in the incidence of PTC in HT. The number of HT patients who develop nodules was not given. Next, we could not estimate the mean time for the HT patient who would suffer from PTC. It is necessary that the basic research on raised TSH causing PTC in HT patients should be done in the future, which could provide more powerful evidence for the correlation of HT and PTC.

## Conclusion

PTC and HT have a close relationship in an area with a high prevalence of HT disease. Based on the results of our study, we hypothesise that long-term HT could cause elevated serum TSH, which is the real risk factor for the development of thyroid cancer. Furthermore, the disease’s invasiveness and metastasis in patients suffering from both PTC and HT could be used as a model in studies investigating inflammation and cancer progression. Additional research should focus on this interesting issue.

## Methods

### Patients

The study subjects were all patients treated from January, 2008, to December, 2010, at the Department of Head and Neck Surgery at the Cancer Hospital of Fudan University in Shanghai, China. All of the patients provided written informed consent for their information to be stored in the hospital database and used for research, and the study was approved by the Ethical Committee of the Cancer Hospital of Fudan University. The patients included in the study met the following criteria: no previous thyroid surgery, no PTC or benign thyroid nodule confirmed by histopathology, and the availability of an adequate medical history. A total of 6109 patients were included in this study.

All patients with thyroid nodules underwent a thyroid US examination in our hospital. In our hospital, we selected patients with thyroid nodules for surgery based on the suspicious features on the ultrasound images, which were defined as micro-calcification, hypo-echoic, increased nodular vascularity, infiltrative margin and taller than wide on transverse view. A suspicious malignant nodule was diagnosed when at least one of the above images was present. Surgical operation was suggested in patients with at least one suspicious malignant nodule.

If more than one nodule were found under US, each nodule in the whole thyroid gland was evaluated by one clinical physician at the same time.

All patients that have not undergone operation were followed with serial US examinations every 3–6 months, especially in those with suspicious malignant nodule but rejected the surgery suggestion considering the relative good prognosis of PTMC and the potential risk of the operation. Surgery was indicated during period of follow-up when there was more than a 50% change in volume of suspicious malignant nodules or presence of at least one suspicious US feature mentioned above in other nodules.

In abstract, there were three criteria for our selecting patients before surgery.

1. suspect ultrasound images, for example, microcalcifications, hypoechoic, increased nodular vascularity, infiltrative margins and taller than wide on transverse view.

2. ultrasound images changed in the 3–6 months follow-up

3. patients consent.

This was described in our previous paper [52]. For patients with benign nodules, surgery only was done in the nodule larger than 4 cm.

### Histopathology

Resected surgical specimens were fixed in 10% formalin and cut at 5-um intervals. One to 3 representative sections of the tumour and all suspicious lesions were submitted for microscopy. All of the sections were fixed in formalin, embedded in paraffin wax, and stained with haematoxylin and eosin. HT was diagnosed by the presence of diffuse lymphoplasmacytic infiltrations with germinal centres, parenchymal atrophy with oncocytic changes, and variable amounts of stromal fibrosis throughout the thyroid gland. We examined and recorded any coexisting HT in all of the patients included in the study.

### Serum thyroid function measurement

In our hospital, serum fT4, fT3, and TSH are measured by a chemiluminescent microparticle immunoassay (Abbott Laboratories, Abbott Park, IL 60064, USA) that has interassay coefficients of variations of less than 10% over the ranges 2.63–5.70 pmol/litre, 9.01–19.05 pmol/litre, and 0.35–4.94 μIU/mlitre.

### Clinicopathologic variables assessed

The following variables were used in our study: gender, age at diagnosis, maximal microscopic tumour size (the largest diameter; for multifocal lesions, the maximal microscopic tumour size of the largest lesion was used, as outlined by the AJCC 7^th^ edition), primary tumour invasion and central lymph node status. Prophylactic CLND was routinely performed when the results of the frozen section indicated a malignant nodule. Invasion of the primary tumour consisted of gross extrathyroidal extension and microscopic extrathyroidal extension, which were assessed in the final pathology. The final pathology decided the status of the lateral neck lymph nodes and if there was central lymph node metastasis.

### Statistical analysis

The results are expressed as the means ± standard deviation (SD). The statistical analysis was performed using a Student’s T test or Mann–Whitney test or chi-square test, when appropriate. ORs with 95% confidence intervals (CIs) for the relationships between the variables and PTC were calculated using binary logistic regression. A p-value of less than 0.05 was considered statistically significant. The statistical analyses were performed using SPSS software, version 12.0 (SPSS, Chicago, IL, USA).

## Abbreviations

PTC: Papillary thyroid cancer; HT: Hashimoto’s thyroiditis; CLNM: Central compartment lymph node metastasis; TSH: Serum thyroid stimulating hormone; fT3: free triiodothyronine; fT4: free thyroxine; LT4: Levothyroxine; OR: Odds rate; CLND: Central lymph node dissection; GWAS: Genome-wide association studies; SD: Standard deviation; CI: Confidence intervals.

## Competing interests

The authors have declared that no competing interests exist.

## Authors’ contributions

LZ and HL had equally made substantial contributions to acquisition of data, analysis and interpretation of data and draft the manuscript for important intellectual content. QJ made contribution to the conception and design of this study; give final approval of the version to be published. YZ, ZW, YW, CH and QS made contributions to acquisition of data. DL and YW made contributions to interpretation of data. All authors read and approved the final manuscript.

## Pre-publication history

The pre-publication history for this paper can be accessed here:

http://www.biomedcentral.com/1471-2407/12/610/prepub

## Supplementary Material

Additional file 1 **Figure S1.** Hashimoto’s thyroiditis. Germinal centers have formed in the gland and are associated with the smaller follicles and the oxyphilic follicular cells. HE x 200. **Figure S2.** Papillary carcinoma arises in the background OF Hashimoto’s thyroiditis. HE x 40. **Figure S3.** PTC co-existence with HT. The carcinoma area (the lower portion) is next to Hashimoto’s thyroiditis area(the upper portion). HE x 200. **Figure S4.** PTC Distinct papillary structures are seen. A giant cell, common in papillary carcinoma, is seen in this section. HE x 200. Click here for file
