# Supplementary material for: The clinical features of papillary thyroid cancer in Hashimoto’s thyroiditis patients from an area with a high prevalence of Hashimoto’s disease
Source: BMC Cancer. 2012 Dec 21;12:610. doi: 10.1186/1471-2407-12-610 (PMC3547693; doi:10.1186/1471-2407-12-610)
Supplement: Additional file 1 — Figure S1. Hashimoto’s thyroiditis. Germinal centers have formed in the gland and are associated with the smaller follicles and the oxyphilic follicular cells. HE x 200. Figure S2. Papillary carcinoma arises in the background OF Hashimoto’s thyroiditis. HE x 40. Figure S3. PTC co-existence with HT. The carcinoma area (the lower portion) is next to Hashimoto’s thyroiditis area(the upper portion). HE x 200. Figure S4. PTC Distinct papillary structures are seen. A giant cell, common in papillary carcinoma, is seen in this section. HE x 200. [file 1471-2407-12-610-S1.doc]

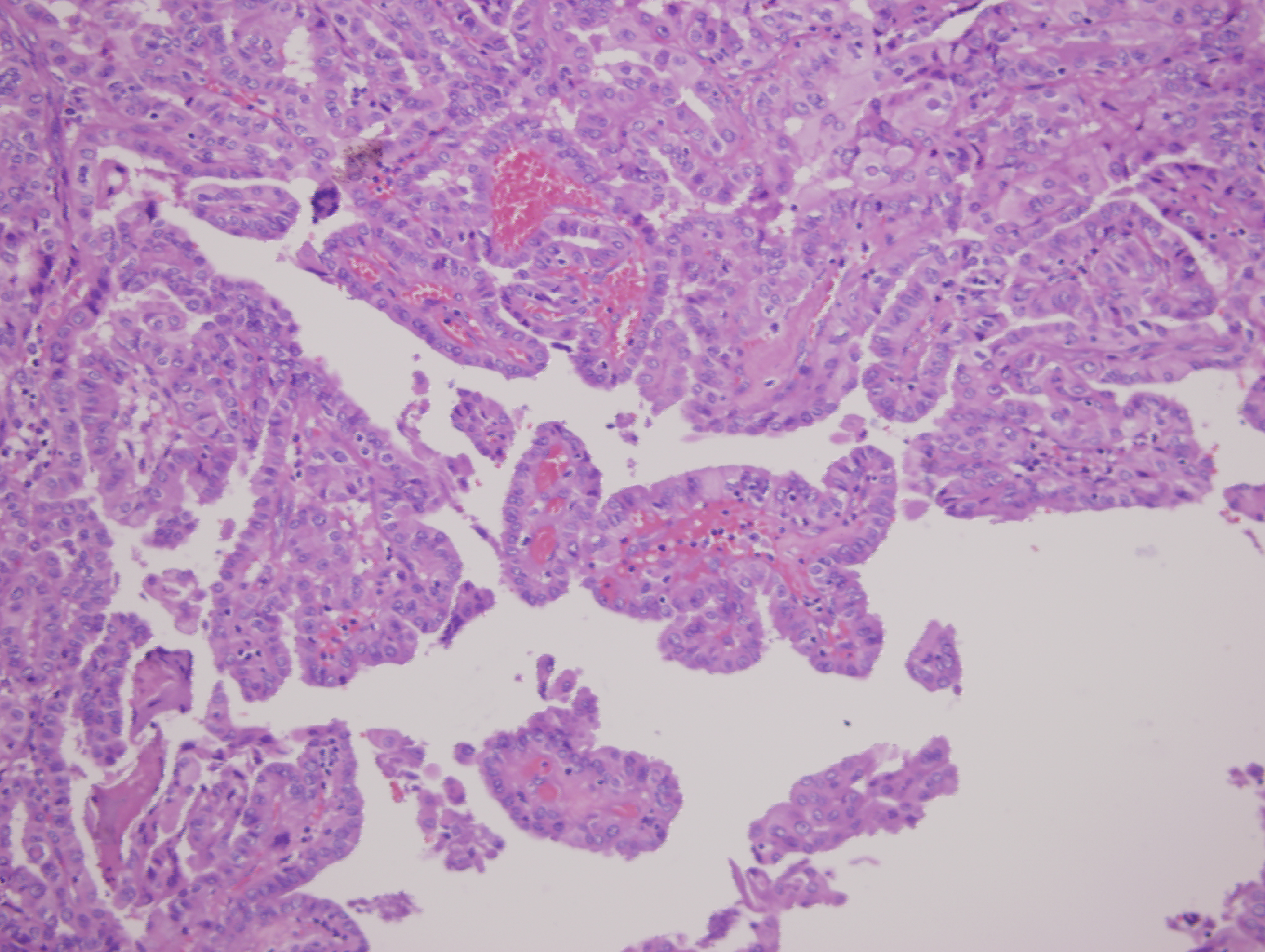


Supplemental Figure S1 Hashimoto’s thyroiditis. Germinal centers have formed in the gland and are associated with the smaller follicles and the oxyphilic follicular cells. HE x 200


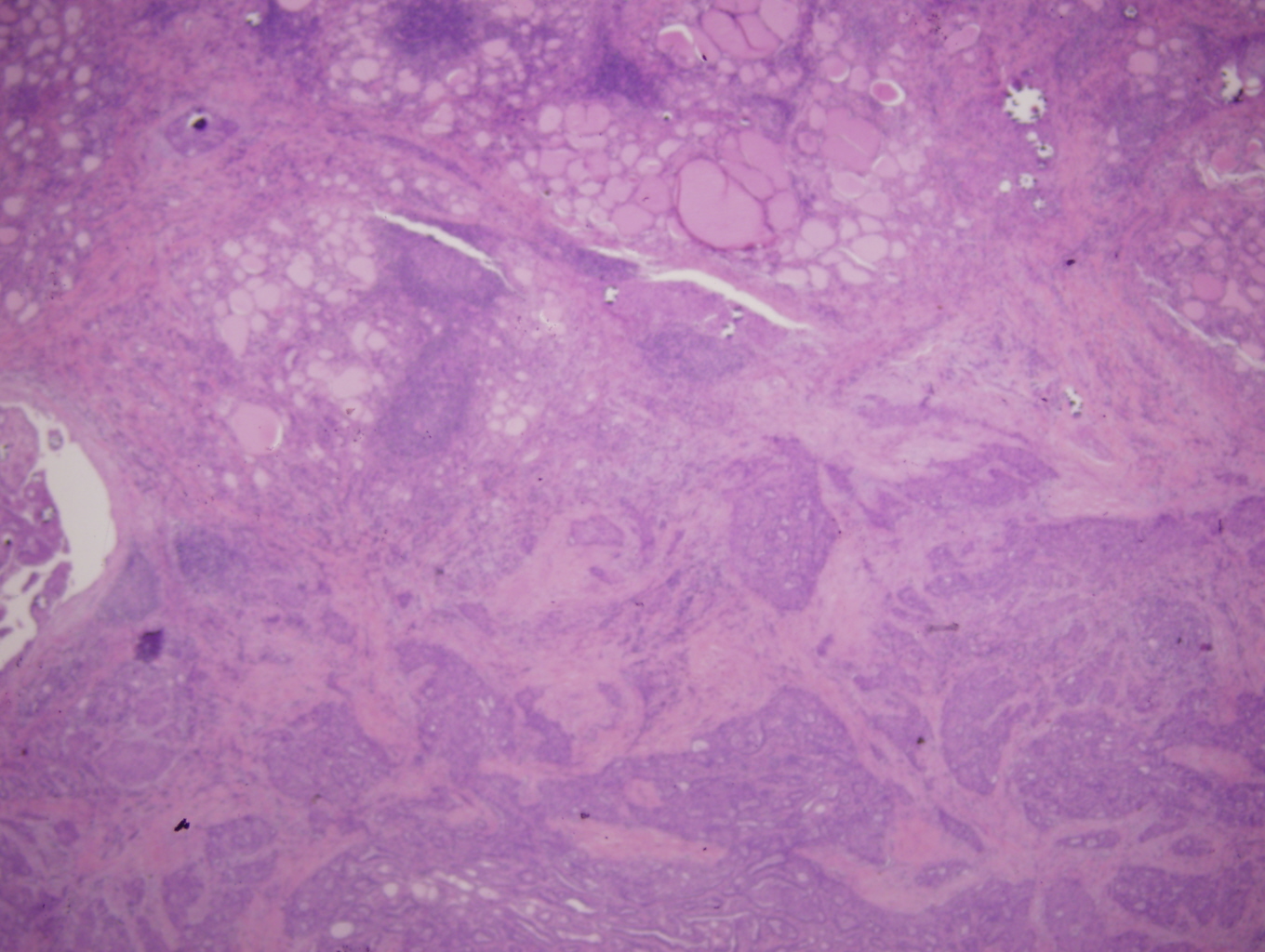


Supplemental Figure S2 Papillary carcinoma arises in the background OF Hashimoto’s thyroiditis. HE x 40


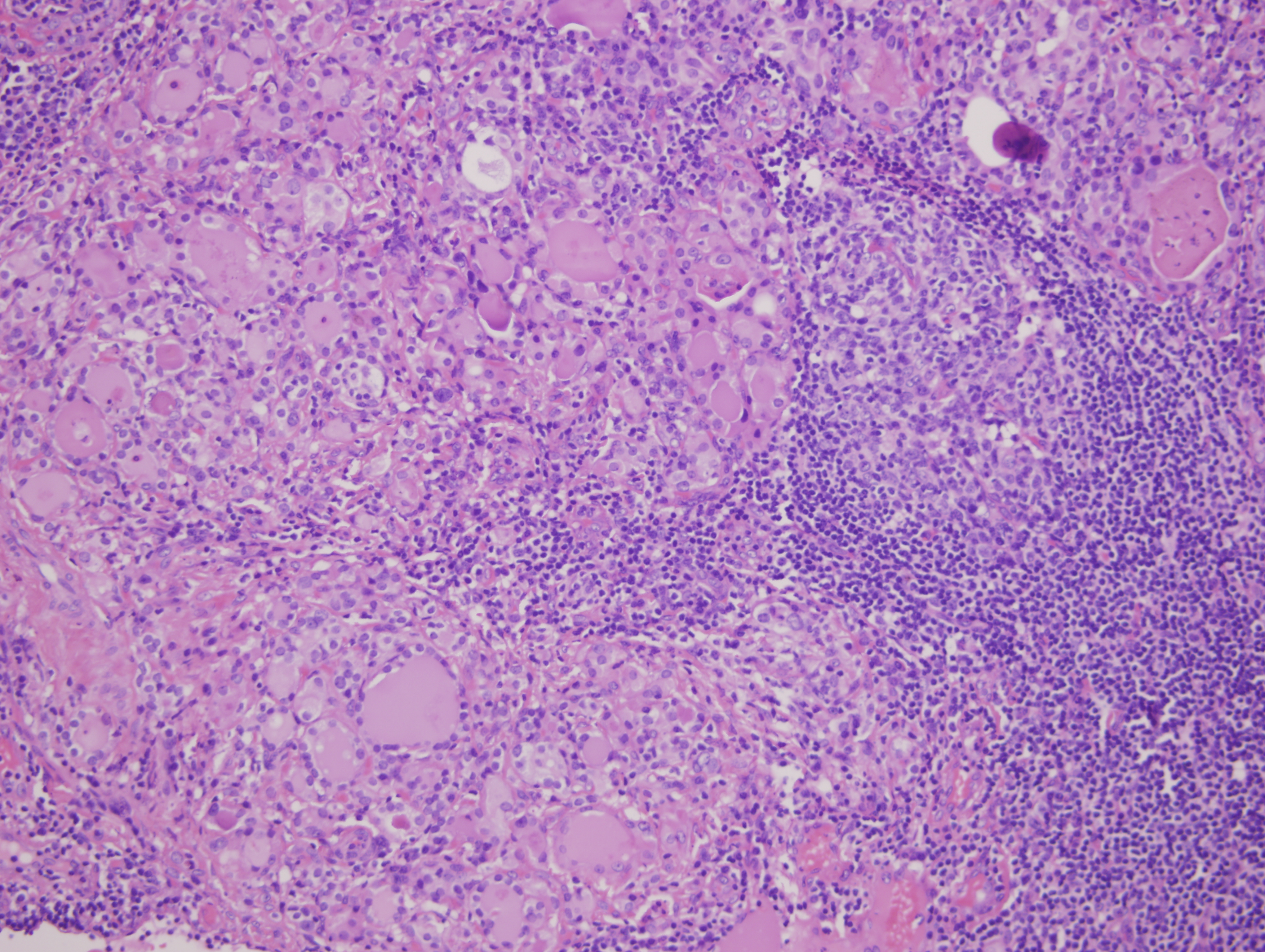


Supplemental Figure S3 PTC co-existence with HT. The carcinoma area (the lower portion) is next to Hashimoto’s thyroiditis area(the upper portion). HE x 200


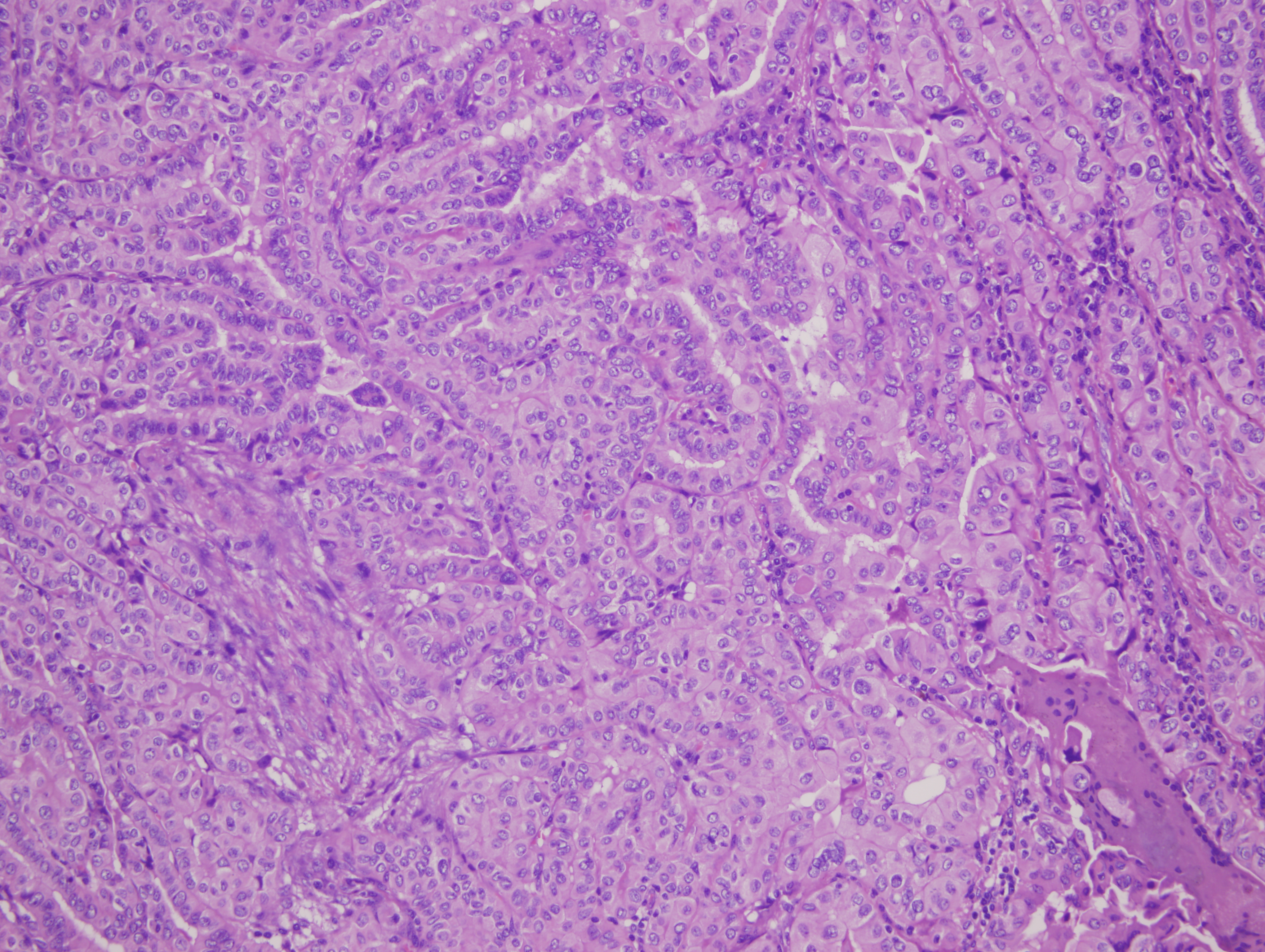


Supplemental Figure S4 PTC Distinct papillary structures are seen. A giant cell , common in papillary carcinoma, is seen in this section. HE x 200
